# *Clostridium difficile* Bacteremia, Taiwan1

**DOI:** 10.3201/eid1608.100064

**Published:** 2010-08

**Authors:** Nan-Yao Lee, Yu-Tsung Huang, Po-Ren Hsueh, Wen-Chien Ko

**Affiliations:** Author affiliations: National Cheng Kung University Hospital and Medical College, Tainan, Taiwan (N.-Y. Lee, W.-C. Ko);; National Taiwan University Hospital and College of Medicine, Taipei, Taiwan (Y.-T. Huang, P.-R. Hsueh)

**Keywords:** Bacteria, Clostridium difficile, bacteremia, enteric infections, outcomes, metronidazole, vancomycin, synopsis

## Abstract

*C. difficile* bacteremia, although uncommon, is associated with severe illness and death.

## MEDSCAPE CME 

Medscape, LLC is pleased to provide online continuing medical education (CME) for this journal article, allowing clinicians the opportunity to earn CME credit. This activity has been planned and implemented in accordance with the Essential Areas and policies of the Accreditation Council for Continuing Medical Education through the joint sponsorship of Medscape, LLC and Emerging Infectious Diseases. Medscape, LLC is accredited by the ACCME to provide continuing medical education for physicians. Medscape, LLC designates this educational activity for a maximum of 0.25 *AMA PRA Category 1 Credits*™. Physicians should only claim credit commensurate with the extent of their participation in the activity. All other clinicians completing this activity will be issued a certificate of participation. To participate in this journal CME activity: (1) review the learning objectives and author disclosures; (2) study the education content; (3) take the post-test and/or complete the evaluation at http://cme.medscape.com/viewpublication/30063; (4) view/print certificate.

## Learning Objectives

Upon completion of this activity, participants will be able to:

- Identify presenting symptoms of *Clostridium difficile* bacteremia (CDB).
- Specify the most common source of bacteremia in cases of CDB and incorporate that knowledge into the development of effective management plans.
- Describe the prognosis of CDB.

## Editor

**Nancy Mannikko, MS, PhD**, Technical Writer/Editor, *Emerging Infectious Diseases. Disclosure: Nancy Mannikko, MS, PhD, has disclosed no relevant financial relationships.*

## CME AUTHOR

**Charles P. Vega, MD**, Associate Professor; Residency Director, Department of Family Medicine, University of California, Irvine. *Disclosure: Charles P. Vega, MD, has disclosed no relevant financial relationships.*

## AUTHORS

*Disclosures:* ***Nan-Yao Lee, MD; Yu-Tsung Huang, MD; Po-Ren Hsueh, MD;*** *and* ***Wen-Chien Ko, MD,*** *have disclosed no relevant financial relationships.*

## References

[1] Aslam S, Hamill RJ, Musher DM. Treatment of *Clostridium difficile*–associated disease: old therapies and new strategies. Lancet Infect Dis. 2005;5:549–57. 10.1016/S1473-3099(05)70215-2 16122678

[2] Rupnik M, Wilcox MH, Gerding DN. *Clostridium difficile* infection: new developments in epidemiology and pathogenesis. Nat Rev Microbiol. 2009;7:526–36. 10.1038/nrmicro2164 19528959

[3] Garcia-Lechuz JM, Hernangomez S, Juan RS, Pelaez T, Alcala L, Bouza E. Extra-intestinal infections caused by *Clostridium difficile.* Clin Microbiol Infect. 2001;7:453–7. 10.1046/j.1469-0691.2001.00313.x 11591212

[4] Jacobs A, Barnard K, Fishel R, Gradon JD. Extracolonic manifestations of *Clostridium difficile* infections. Presentation of 2 cases and review of the literature. Medicine (Baltimore). 2001;80:88–101. 10.1097/00005792-200103000-00002 11307591

[5] Byl B, Jacobs F, Struelens MJ, Thys JP. Extraintestinal *Clostridium difficile* infections. Clin Infect Dis. 1996;22:712. 10.1093/clinids/22.4.712 8729213

[6] Libby DB, Bearman G. Bacteremia due to *Clostridium difficile*–review of the literature. Int J Infect Dis. 2009;13:e305–9. 10.1016/j.ijid.2009.01.014 19398213

[7] Saginur R, Fogel R, Begin L, Cohen B, Mendelson J. Splenic abscess due to *Clostridium difficile.* J Infect Dis. 1983;147:1105. 10.1093/infdis/147.6.1105 6854068

[8] Rampling A, Warren RE, Bevan PC, Hoggarth CE, Swirsky D, Hayhoe FG. *Clostridium difficile* in haematological malignancy. J Clin Pathol. 1985;38:445–51. 10.1136/jcp.38.4.445 3857233 PMC499176

[9] Feldman RJ, Kallich M, Weinstein MP. Bacteremia due to *Clostridium difficile*: case report and review of extraintestinal *C. difficile* infections. Clin Infect Dis. 1995;20:1560–2. 10.1093/clinids/20.6.1560 7548512

[10] Wolf LE, Gorbach SL, Granowitz EV. Extraintestinal *Clostridium difficile*: 10 years’ experience at a tertiary-care hospital. Mayo Clin Proc. 1998;73:943–7. 10.4065/73.10.943 9787741

[11] McCabe WR, Jackson GG. Gram-negative bacteremia: I. Etiology and ecology. Arch Intern Med. 1962;110:847–55. 10.1001/archinte.1962.03620240029006

[12] Charlson ME, Pompei P, Ales KL, MacKenzie CR. A new method of classifying prognostic comorbidity in longitudinal studies: development and validation. J Chronic Dis. 1987;40:373–83. 10.1016/0021-9681(87)90171-8 3558716

[13] Knaus WA, Draper EA, Wagner DP, Zimmerman JE. APACHE II: a severity of disease classification system. Crit Care Med. 1985;13:818–29. 10.1097/00003246-198510000-00009 3928249

[14] Le Gall JR, Lemeshow S, Saulnier F. A new Simplified Acute Physiology Score (SAPS II) based on a European/North American multicenter study. JAMA. 1993;270:2957–63. 10.1001/jama.1993.03510240069035 8254858

[15] Yu VL, Chiou CC, Feldman C, Ortqvist A, Rello J, Morris AJ, An international prospective study of pneumococcal bacteremia: correlation with in vitro resistance, antibiotics administered, and clinical outcome. Clin Infect Dis. 2003;37:230–7. 10.1086/377534 12856216

[16] Bone RC, Balk RA, Cerra FB, Dellinger RP, Fein AM, Knaus WA, Definitions for sepsis and organ failure and guidelines for the use of innovative therapies in sepsis. The ACCP/SCCM Consensus Conference Committee. American College of Chest Physicians/Society of Critical Care Medicine. Chest. 1992;101:1644–55. 10.1378/chest.101.6.1644 1303622

[17] Garner JS, Jarvis WR, Emori TG, Horan TC, Hughes JM. CDC definitions for nosocomial infections, 1988. Am J Infect Control. 1988;16:128–40. 10.1016/0196-6553(88)90053-3 2841893

[18] Baron EJ, Weissfeld AS, Fuselier PA, Brenner DJ. Classification and identification of bacteria. In: Murray PR, Baron EJ, Pfaller MA, Tenover FC, Yolken RH, editors. Manual of clinical microbiology, 6th ed. Washington: American Society for Microbiology Press; 1995. p. 249–64.

[19] Persson S, Torpdahl M, Olsen KE. New multiplex PCR method for the detection of *Clostridium difficile* toxin A (*tcdA*) and toxin B (*tcdB*) and the binary toxin (*cdtA/cdtB*) genes applied to a Danish strain collection. Clin Microbiol Infect. 2008;14:1057–64. 10.1111/j.1469-0691.2008.02092.x 19040478

[20] Klaassen CH, van Haren HA, Horrevorts AM. Molecular fingerprinting of *Clostridium difficile* isolates: pulsed-field gel electrophoresis versus amplified fragment length polymorphism. J Clin Microbiol. 2002;40:101–4. 10.1128/JCM.40.1.101-104.2002 11773100 PMC120100

[21] Spigaglia P, Mastrantonio P. Evaluation of repetitive element sequence-based PCR as a molecular typing method for *Clostridium difficile.* J Clin Microbiol. 2003;41:2454–7. 10.1128/JCM.41.6.2454-2457.2003 12791864 PMC156551

[22] Clinical and Laboratory Standards Institute. Methods for antimicrobial susceptibility testing of anaerobic bacteria. Approved standard, 7th ed. M11–A7. Wayne (PA): The Institute; 2007.

[23] Bedimo R, Weinstein J. Recurrent extraintestinal *Clostridium difficile* infection. Am J Med. 2003;114:770–1. 10.1016/S0002-9343(03)00184-0 12829209

[24] Cid A, Juncal AR, Aguilera A, Regueiro BJ, Gonzalez V. *Clostridium difficile* bacteremia in an immunocompetent child. J Clin Microbiol. 1998;36:1167–8. 9542965 10.1128/jcm.36.4.1167-1168.1998 PMC104717

[25] Genta VM, Gilligan PH, McCarthy LR. *Clostridium difficile* peritonitis in a neonate. A case report. Arch Pathol Lab Med. 1984;108:82–3. 6546347

[26] Studemeister AE, Beilke MA, Kirmani N. Splenic abscess due to *Clostridium difficile* and *Pseudomonas paucimobilis.* Am J Gastroenterol. 1987;82:389–90. 3565349

[27] Spencer RC, Courtney SP, Nicol CD. Polymicrobial septicaemia due to *Clostridium difficile* and *Bacteroides fragilis.* Br Med J (Clin Res Ed). 1984;289:531–2. 10.1136/bmj.289.6444.531 6432176 PMC1442715

[28] Smith LD, King EO. Occurrence of *Clostridium difficile* in infections of man. J Bacteriol. 1962;84:65–7. 13914327 10.1128/jb.84.1.65-67.1962 PMC277766

[29] Gerard M, Defresne N, Van der Auwera P, Meunier F. Polymicrobial septicemia with *Clostridium difficile* in acute diverticulitis. Eur J Clin Microbiol Infect Dis. 1989;8:300–2. 10.1007/BF01963455 2497002

[30] Elliott B, Reed R, Chang BJ, Riley TV. Bacteremia with a large clostridial toxin-negative, binary toxin-positive strain of *Clostridium difficile.* Anaerobe. 2009;15:249–51. 10.1016/j.anaerobe.2009.08.006 19723585

[31] Benjamin B, Kan M, Schwartz D, Siegman-Igra Y. The possible significance of *Clostridium* spp. in blood cultures. Clin Microbiol Infect. 2006;12:1006–12. 10.1111/j.1469-0691.2006.01464.x 16961638

[32] Rechner PM, Agger WA, Mruz K, Cogbill TH. Clinical features of clostridial bacteremia: a review from a rural area. Clin Infect Dis. 2001;33:349–53. 10.1086/321883 11438901

[33] Pietrafitta JJ, Deckers PJ. Significance of clostridial bacteremia. Am J Surg. 1982;143:519–22. 10.1016/0002-9610(82)90206-9 6280507

[34] Balzan S, de Almeida Quadros C, de Cleva R, Zilberstein B, Cecconello I. Bacterial translocation: overview of mechanisms and clinical impact. J Gastroenterol Hepatol. 2007;22:464–71. 10.1111/j.1440-1746.2007.04933.x 17376034

[35] Chen YM, Lee HC, Chang CM, Chuang YC, Ko WC. *Clostridium* bacteremia: emphasis on the poor prognosis in cirrhotic patients. J Microbiol Immunol Infect. 2001;34:113–8. 11456356

[36] Kelly CP, Pothoulakis C, LaMont JT. *Clostridium difficile* colitis. N Engl J Med. 1994;330:257–62. 10.1056/NEJM199401273300406 8043060

[37] Salonen JH, Eerola E, Meurman O. Clinical significance and outcome of anaerobic bacteremia. Clin Infect Dis. 1998;26:1413–7. 10.1086/516355 9636872

[38] Bartlett JG. Narrative review: the new epidemic of *Clostridium difficile*–associated enteric disease. Ann Intern Med. 2006;145:758–64. 17116920 10.7326/0003-4819-145-10-200611210-00008

