# Supplementary material for: Clostridium difficile Bacteremia, Taiwan1
Source: Emerg Infect Dis. 2010 Aug;16(8):1204–10. doi: 10.3201/eid1608.100064 (PMC3298294; doi:10.3201/eid1608.100064)
Supplement: Related texts

# Review article: *Clostridium difficile* Bacteremia, Taiwan^1^

**DOI:** 10.3201/eid1608.100064

**Authors:** Nan-Yao Lee, Yu-Tsung Huang, Po-Ren Hsueh, Wen-Chien Ko

**Affiliations:** Author affiliations: National Cheng Kung University Hospital and Medical College, Tainan, Taiwan (N.-Y. Lee, W.-C. Ko);; National Taiwan University Hospital and College of Medicine, Taipei, Taiwan (Y.-T. Huang, P.-R. Hsueh)

## Abstract

To determine clinical characteristics and outcome of patients with *Clostridium difficile* bacteremia (CDB), we identified 12 patients with CDB in 2 medical centers in Taiwan; all had underlying systemic diseases. Five had gastrointestinal diseases or conditions, including pseudomembranous colitis (2 patients); 4 recalled diarrhea, but only 5 had recent exposure to antimicrobial drugs. Ten available isolates were susceptible to metronidazole and vancomycin. Five isolates had *C. difficile* toxin A or B. Of 5 patients who died, 3 died of CDB. Of 8 patients treated with metronidazole or vancomycin, only 1 died, and all 4 patients treated with other drugs died (12.5% vs. 100%; p = 0.01). *C. difficile* bacteremia, although uncommon, is thus associated with substaintial illness and death rates.

*Clostridium difficile* is well recognized as the etiologic agent of pseudomembranous colitis and has been implicated as the cause of 10%–25% of cases of antimicrobial drug–associated diarrhea (*1*). The pathogen has been responsible for numerous recent hospital-based epidemics and is also emerging in the community (*2*). The clinical features, disease spectrum and pathogenesis, and optimal treatments of *C. difficile*–associated diarrhea have been well studied. In contrast, reports of the isolation of *C. difficile* in body sites other than the intestines, or extraintestinal infections, have been anecdotal (*3**,**4*). Extracolonic manifestations of *C. difficile* infections reported were variable, including bacteremia, osteomyelitis, visceral abscess, empyema, reactive arthritis, pyelonephritis, prosthetic joint infection, and skin and soft tissue infection (*3**–**10*). Most cases of extracolonic *C. difficile* infections have been preceded by gastrointestinal events, either *C. difficile* colitis or surgical and anatomic disruption of the colon (*4*).

Recently, Libby and Bearman reviewed the literature on bacteremia caused by *C. difficile* (*6*). Most cases were identified from individual case reports. However, as the incidence of *C. difficile* infection increases, an increase in cases of *C. difficile* bacteremia (CDB) is likely (*10*). Knowledge of the clinical signs and symptoms of these extracolonic manifestations of bloodstream infections will be useful in patient care and could improve clinical outcomes (*4*). To outline the spectrum and clinical significance of CDB, we report 12 cases of CDB over a recent 10-year period at 2 medical centers in Taiwan and review the literature published in English.

## Patients

Patients with blood cultures positive for *C. difficile* at 2 teaching hospitals (National Cheng Kung University Hospital, a 1,100-bed tertiary care hospital in southern Taiwan and National Taiwan University Hospital, a 2,800-bed tertiary care hospital in northern Taiwan) during January 1989 through February 2009, were retrospectively identified from laboratory records, and their medical records were reviewed. If a patient experienced >1 episode of CDB, only information about the first episode was included. Information about age, sex, underlying diseases, clinical course, antimicrobial drug therapy, and clinical outcome was recorded in a case-record form.

We conducted a literature review to find relevant articles published between January 1, 1962, and August 31, 2009, by querying the PubMed database using the keywords “*Clostridium difficile,*” “bacteremia,” “sepsis,” and “bloodstream infection.” The references of available articles were surveyed for additional cases.

### Definitions

Underlying conditions were stratified on the basis of the McCabe and Jackson score (*11*) and the comorbidity score of Charlson et al. (*12*). Severity of illness was evaluated on the first day of bacteremia onset by means of the Acute Physiology and Chronic Health Evaluation II Score (*13*), Simplified Acute Physiology Score II (*14*), and the Pittsburgh bacteremia score (*15*). The physiologic response to bacteremia was categorized as severe sepsis if the patient met the criteria for severe sepsis specified by the American College of Chest Physicians/Society for Critical Care Medicine Consensus Conference Committee (*16*). Immunosuppressive therapy was defined as the receipt of corticosteroid treatment (10 mg/day or an equivalent dosage) for >2 weeks of antineoplastic chemotherapy or antirejection medication within 30 days before admission. Sepsis-related death was defined as the death of a patient with a clinical course that suggested persistently active infection if the patient had no other obvious explanation for the death.

Bacteremia was defined as the presence of an organism in a blood culture specimen. Clinically significant bacteremia was defined as >1 positive blood culture and clinical features compatible with fever and sepsis syndrome; patients with noteworthy bacteremia were included in this study. An episode of bacteremia was considered to be hospital acquired if a bacteremic episode was noted at >48 hours after hospitalization; healthcare associated if it occurred within 48 hours of hospitalization in patients with extensive contact with the healthcare system (such as nursing home residence, organ transplantation, hemodialysis dependence, presence of an indwelling intravascular catheter, or surgery within the previous 30 days), or the patients had been transferred from another hospital or long-term care facility; or community acquired if it occurred <48 hours of admission in patients without extensive healthcare contact. In the case of secondary bacteremia, a primary focus of infection was defined according to the criteria of the Centers for Disease Control and Prevention (*17*).

Antimicrobial drug therapy in the preceding 30 days was documented through a review of medical records. Previous antimicrobial drug therapy was defined as the receipt of an oral or parenteral antimicrobial agent for >72 hours within the preceding 2 months.

### Identification of Isolates and Clonality

Bacteria colonies suspected of being *C. difficile* on the basis of characteristic odor, typical morphologic features, and Gram staining results were phenotypically identified by using standard methods (*18*) and the API 32A system (bioMérieux, Las Halles, France). These organisms were further confirmed to the species level by using partial 16S rRNA gene sequence analysis. Two primers were used: PS13 5′-GGAGGCAGCAGTGGGGAATA-3′ and PS14 5′-TGACGGGCGGTGTGTACAAG-3′, as described (*19*). The partial sequences obtained (529 bp) were compared with published sequences in the GenBank database by using the BLASTN algorithm (www.ncbi.nlm.nih.gov/blast). Molecular typing of these isolates by pulsed-field gel electrophoresis (PFGE) using *Sma*I (New England Biolabs, Beverly, MA, USA)–digested DNA and repetitive-element PCR typing (DiversiLab Kits for *C. difficile*; bioMérieux) were performed to identify the clonality of the isolates (*20**,**21*). For PFGE, gels were run for 22 h at 13°C by using a CHEF DR-III system (Bio-Rad Laboratories, Hercules, CA, USA) at 5.5 V/cm with initial and final pulse times of 5 s and 60 s, respectively (*20*). The production of *C. difficile* toxin A or B was detected by a qualitative enzyme immunoassay (Premier toxin A&B; Meridian Bioscience, Inc., Cincinnati, OH, USA).

### Antimicrobial Drug Susceptibility Testing

MICs of 14 antimicrobial drugs (penicillin, piperacillin/tazobactam, ampicillin/sulbactam, cefmetazole, meropenem, imipenem, doripenem, ertapenem, metronidazole, vancomycin, teicoplanin, fusidic acid, clindamycin, and daptomycin) were determined by the agar dilution method described by the Clinical and Laboratory Standards Institute (*22*). MICs of tigecycline were measured by the broth microdilution method. The antimicrobial agents used for susceptibility testing were obtained from their corresponding manufacturers. The MIC breakpoints followed those recommended by the Clinical and Laboratory Standards Institute (*22*) or the European Committee on Antimicrobial Susceptibility Testing (www.eucast.org).

### Statistical Analysis

The results were analyzed with SPSS software for Windows, version 13.0 (SPSS, Chicago, IL, USA). Continuous variables were expressed as mean ± SD, and categorical variables were expressed as percentages of total number of specific patients analyzed. Categorical variables were compared by the Fisher exact test or χ^2^ test, as appropriate. Continuous variables were compared by the Mann–Whitney U or Student *t* test. All tests for statistical significance were 2-tailed, and p values <0.05 were considered significant.

Twelve patients, 7 from National Taiwan University Hospital and 5 from National Cheng Kung University Hospital, with CDB during the period of the study were identified. Most (*11*) patients were identified after 2000. The partial 16S rRNA gene sequence analysis of 10 available isolates identified by conventional methods showed that they most closely matched *C. difficile* isolates (accession number FN545816.1; maximal identity: 100% [529/529]). Only 10 isolates were available, and the earliest isolate was obtained in 2005. Their genotyping patterns determined by the repetitive-element PCR and PFGE were different, indicating that no intrahospital or interhospital spread of *C. difficile* isolates occurred in the 2 hospitals*.*

The epidemiologic and clinical data of the 12 patients with *C. difficile* bacteremia are shown in Table 1 and Table 2. Their mean ± SD age was 59.9 ± 22.1 years (range 12–87 years). Women (7 cases) outnumbered men. All patients had chronic medical illnesses, particularly diabetes mellitus (*4*) and cirrhosis of the liver (*6*). Eleven patients had no underlying or rapidly fatal diseases, according to the McCabe and Bearman classification, and the mean comorbidity score of Charlson et al. was 5.2 (SD 2.2). At the time of bacteremia onset, the mean Acute Physiology And Chronic Health Evaluation II Score or Simplified Acute Physiology Score II score was 25.4 (SD 0.9) or 50.0 (SD 25.2), respectively. Seven patients had critical illness defined as having at least 4 points of the Pittsburgh bacteremia score (*15*). Five patients were considered to have community-onset CDB, but all had a history of recent hospitalization or referral from a chronic care facility and thus all of their infections can be classified as healthcare associated. . The average length of hospital stay before CDB was 16.4 days. Primary bacteremia noted in 7 patients was the most common infection noted, followed by intraabdominal infections (4, 33%), bone and joint infections (1, 8.3%), and skin and soft-tissue infections (1, 8.3%).

**Table 1:** Clinical manifestations, antimicrobial drug therapy, and outcome of 12 patients with *Clostridium difficile* bacteremia, Taiwan, 1989–2009*

Patient no.	Age, y/sex	Clinical signs and symptoms	Sources of bacteremia	Coexisting condition(s)	Copathogen(s)	Clostridial toxin assay result	Treatment/ outcome
Monomicrobial bacteremia							
1	69/F	Dead on arrival	Primary bacteremia	Liver cirrhosis	None	Positive	None/died
2	38/M	Abdominal pain	IAI (primary peritonitis)	Wilson disease	None	Negative	Cefmetazole for 22 d/died
3	65/F	Fever, abdominal pain	IAI (secondary peritonitis)	Perforated peptic ulcer with exploratory laparotomy	None	NA	Metronidazole† for 10 d/died
4	58/M	Fever, abdominal pain	IAI (primary peritonitis)	Liver cirrhosis	None	Negative	Metronidazole† for 12 d/survived
5	12/M	Fever, dyspnea	Primary bacteremia	Biliary atresia, liver transplantation	None	Negative	Piperacillin- tazobactam and vancomycin† for 15 d/survived
6	41/F	Fever, dyspnea	Primary bacteremia	Pulmonary fibrosis	None	Negative	Ceftazidime and gentamicin for 13 d; vancomycin† for 10 d/survived
Polymicrobial bacteremia							
7	45/M	Abdominal pain	Primary bacteremia	Liver cirrhosis	Coagulase- negative *Staphylococcus* spp.	Positive	Ceftriaxone for 3 d/died
8	83/M	Fever, bloody stool	Primary bacteremia	Gastrointestinal bleeding, hypovolemic shock	*Escherichia coli*	Negative	Imipenem for 1 d/died
9	87/F	Bloody stool	Primary bacteremia	Congestive heart failure, end-stage renal disease, pseudomembranous colitis	*Pseudomonas aeruginosa*, *Enterococcus faecium*, *E. coli*, ESBL–*Klebsiella oxytoca*	Positive	Vancomycin† and meropenem for 7 d/survived
10	80/F	Bloody stool	Primary bacteremia	Liver cirrhosis, pseudomembranous colitis	Coagulase- negative *Staphylococcus* spp.	Positive	Metronidazole† for 13 d/survived
11	66/F	Fever, lower gastrointestinal bleeding, abdominal pain	SSTI/septic arthritis	Femoral neck fracture (received total hip replacement with prosthetic infections), chronic kidney disease	*Enterobacter cloacae*	Negative	Debridement, cefepime and metronidazole† for 12 d/survived
12	75/F	Fever, chills, nausea, vomiting, abdominal pain	IAI (primary peritonitis)	Lymphoma, biliary tract infection	*K. pneumoniae*, *Clostridium perfringens*	NA	Cefepime for 10 d and metronidazole† for 7 d/survived

* IAI, intra-abdominal infection; NA, not available; SSTI, skin and soft tissue infection; ESBL, extended-spectrum β-lactamase. †In vitro active against *C. difficile*.

**Table 2:** Summary of clinical characteristics of 12 patients with *Clostridium difficile* bacteremia in the current series from Taiwan, 1989–2009, and of 20 additional cases published since 1962*

Characteristic	Total, n = 32	Reported cases, n = 20	Current series, n = 12	p value
Age, y, mean ± SD	51.4 ± 26.1	46.2 ± 27.5	59.9 ± 22.2	0.14
Elderly, age >60 y	18 (56.3)	11 (55.0)	7 (58.3)	1.0
Male	19 (59.4)	14 (70.0)	5 (41.7)	0.15
Place of acquisition				0.70
Community	12/28 (42.9)	5/16 (73.3)	7 (58.3)	
Hospital	16/28 (57.1)	11/16 (26.7)	5 (41.7)	
Calendar year range				0.001
1962–1990	8 (25)	8 (40.0)	0	
1991–2000	8 (25)	7 (35.0)	1 (8.3)	
After 2000	16 (50)	5 (25.0)	11 (91.7)	
Comorbidity				
Malignancy	9 (28.1)	6 (30.0)	3 (25.0)	1.0
Congestive heart failure	4 (12.5)	3 (15.0)	1 (8.3)	1.0
Immunosuppression	7 (21.9)	2 (10.0)	5 (41.7)	0.07
Chronic obstructive pulmonary disease	3 (9.4)	2 (10.0)	1 (8.3)	1.0
Chronic kidney disease	3 (9.4)	1 (5.0)	2 (16.7)	0.54
Liver cirrhosis	7 (21.9)	1 (5.0)	6 (50.0)	0.006
Cerebrovascular accident	2 (6.3)	1 (5.0)	1 (8.3)	1.0
Diabetes mellitus	4 (12.5)	0	4 (33.3)	0.014
Organ transplant	2 (6.3)	0	2 (16.6)	0.13
None	6 (18.8)	6 (30.0)	0	0.06
Clinical signs and symptoms				
Fever	19/27 (70.4)	10/15 (66.7)	9 (75.0)	0.70
Abdominal pain	14/29 (48.3)	8/17 (47.1)	6 (50.0)	1.0
Diarrhea	12/28 (42.9)	8/16 (50.0)	4 (33.3)	0.46
Gastrointestinal disease or condition	10/28 (35.7)	5/16 (31.3)	5 (41.7)	0.7
Gastrointestinal bleeding	5/27 (18.5)	0/15 (0.0)	5 (41.7)	0.01
Gastrointestinal perforation	3/28 (10.7)	2/16 (12.5)	1 (8.3)	1.0
Pseudomembranous colitis	5/27 (18.5)	3/15 (20.0)	2 (16.7)	1.0
Recent antimicrobial drug exposure	18/26 (69.2)	13/14 (92.9)	5 (41.7)	0.009
Sources of bacteremia				
Primary	17 (53.1)	10 (50.0)	7 (58.3)	0.73
Intraabdominal infection	11 (34.4)	7 (35.0)	4 (33.3)	1.0
Urosepsis	2 (6.3)	2 (10.0)	0	0.52
Skin and soft tissue infection	2 (6.3)	1 (5.0)	1 (8.3)	1.0
Bone and joint infection	1 (3.1)	0	1 (8.3)	0.38
Polymicrobial bacteremia	20 (62.5)	14 (70.0)	6 (50.0)	0.29
Positive clostridial toxin assay result	14/22 (63.6)	9/12 (75.0)	5/10 (50.0)	0.42
Crude death rate	15/31 (48.4)	10/19 (52.6)	5 (41.7)	0.72

*Values are no. patients (%) or no. patients/total no. patients evaluated (%), except for age. Patients may have >1 morbidity, clinical sign or symptom, and gastrointestinal disease or condition.

When patients were first seen, their signs and symptoms included fever (9, 75%), abdominal pain (6, 50%), and leukocytosis, which was defined as a leukocyte count >12,000 cells/μL (7, 58.3%). Of note, 5 (41.7%) patients had gastrointestinal disorders or conditions at the time of bacteremia onset. Four (33.3%) had a documented episode of diarrhea before CDB, and 2 patients with diarrhea had endoscopically documented pseudomembranous colitis. Five (41.7%) patients had recent exposure to antimicrobial drugs. Six patients had concurrent bacteremia caused by pathogens other than *C. difficile* (Table 2).

Five (50%) of 10 available isolates showed a positive result for the *C. difficile* toxin assay. All isolates were susceptible to metronidazole and vancomycin (Table 3). Other antimicrobial agents demonstrated variable in vitro antibacterial activity against *C. difficile* isolates. In contrast to imipenem, ertapenem, and doripenem, meropenem showed excellent in vitro activity with a narrow MIC range (0.12 μg/mL–2 μg/mL). Of these isolates, 90% of 10 isolates were resistant to penicillin, and 30% were resistant to clindamycin. Tigecycline and daptomycin, 2 newly marketed drugs for which a recommended breakpoint for *C. difficile* has not yet been determined, show favorable antibacterial activity with low 90% MIC values, 0.06 μg/mL and 2 μg/mL, respectively.

**Table 3:** In vitro antimicrobial drug susceptibilities of 10 bacteremic *Clostridium difficile* isolates, Taiwan, 1989–2009*

Antimicrobial agent	MIC, μg/mL			Resistance breakpoint, μg/mL	No. (%) resistant isolates
	Range	MIC_50_	MIC_90_		
Vancomycin†	0.5–1	0.5	1	>2	0
Metronidazole	0.25–8	1	4	>32	0
Clindamycin	0.03–>256	4	>256	>8	3 (30)
Penicillin	0.25–8	4	4	>2	9 (90)
Ampicillin/sulbactam	0.5–8	2	4	>32/16	0
Cefmetazole	0.25–32	16	32	>64	0
Meropenem	0.12–2	1	2	>16	0
Imipenem	0.12–16	8	16	>16	4 (40)
Ertapenem	0.06–16	8	8	>16	0
Doripenem	0.12–8	4	4	NA	NA
Daptomycin	0.12–2	0.5	2	NA	NA
Tigecycline	0.03–0.12	0.03	0.06	NA	NA
Fusidic acid	1–8	1	2	NA	NA

*MIC_50_, 50% MIC; MIC_90_, 90% MIC, NA, not available. †Vancomycin MIC breakpoint was recommended by the European Committee on Antimicrobial Susceptibility Testing (www.eucast.org); otherwise by the Clinical and Laboratory Standards Institute (*22*).

Before microbiologic information was available, 6 patients received antimicrobial therapy, metronidazole (3 patients), vancomycin (*2*), or cefmetazole (*1*). Overall, only 1 of 8 patients definitively treated with metronidazole or vancomycin died; in contrast, all 4 patients treated by drugs other than metronidazole or vancomycin died (p = 0.01). Of 5 patients who died, 3 died directly of CDB. Seven patients survived and were discharged, and 1 patient was treated surgically for septic prosthetic hip arthritis. The survivors remained in the hospital for a mean of 28 days (range 12–47 days) after diagnosis of CDB.

### Literature Review

Twenty reports of patients with CDB have been published in English in the literature since 1962 (*3**,**5**–**10**,**23**–**30*). The epidemiologic and clinical data are summarized in Table 2. Most (14) patients had polymicrobial bacteremia; concomitant gastrointestinal diseases or conditions of varying severity were noted in 15 patients. Excluding a patient with unknown outcome, 10 of 19 patients died in the hospital. The results of stool toxin assay were available for 12 patients, and clostridial toxin A or B could be detected in 8 patients. Drug information was available for 16 patients. Seven patients (3 of whom died) were treated with metronidazole, and 7 were treated with vancomycin (3 of these patients died). The crude death rate for these published cases was similar to that in the present study (10/19, 52.6% vs. 5/12, 41.7%; p = 0.72).

## Conclusions

A clear clinical picture of *C. difficile* bacteremia has been highlighted in this study: a rare extracolonic *C. difficile* infection with severe illness and high death rates in persons with chronic medical illness. However, the case-patients with CDB had several remarkable clinical characteristics, compared with those of patients with *C. difficile*–associated diarrhea. First, only 64% of 22 bacteremic *C. difficile* isolates had toxin A or B, the virulence factors of *C. difficile*–associated diarrhea. Second, bacteremic patients did not commonly have diarrhea before or at admission to the hospital, and in patients with CDB, a history of recent antimicrobial drug exposure (a common predisposing factor of *C. difficile*–associated diarrhea) was rarely recognized.

Because *Clostridium* species are inhabitants of the gastrointestinal tract, they compromise the integrity of the gastrointestinal tract and may lead to translocation with bacteremia. This translocation is supported by the fact that 75% of 32 patients had certain gastrointestinal symptoms or disorders, which may predispose to CDB either by direct extension through perforation or by promoting translocation of the organism across the intestinal wall (*10*). These findings are consistent with the literature, in which *C. difficile* bacteremia is typically associated with underlying gastrointestinal pathology and frequently occurs as a mixed infection with other gut flora (*6**,**31**,**32*).

*Clostridium* species have been shown to enhance the pathogenicity of other bacteria in mixed infections (*10*), and even monomicrobial *Clostridium* bacteremia has been associated with a high death rate in 2 previous series (*10**,**33*). Half of the patients in the current series had polymicrobial bacteremia. The portal of entry of *Clostridium* species in cirrhotic cases is usually obscure, and the gastrointestinal tract is the most likely source (*34**,**35*). Translocation of intestinal bacteria is the major mechanism for the production of bloodstream infection (*34*). In our study, all patients had >1 preexisting illness, and most (75%) experienced gastrointestinal diseases or conditions. Substantial damage to normal mucosal barriers may provide a portal for anaerobes (*32**,**35*), which suggests that patients with gastrointestinal disorders, such as gastrointestinal bleeding and perforation or advanced liver failure, would be susceptible to *C. difficile* bacteremia. Primary bacteremia accounted for more than half (17, 53.1%) of the 32 cases of CDB. The likelihood of undiagnosed *C. difficile* colitis in these patients seems to be minimal given the absence of diarrhea and radiographic manifestation of colitis shown on computed tomographic scan. Subsequently, the resultant monomicrobial bacteremia was likely secondary to bacterial translocation in the setting of immunologic deficiency from an overwhelming preexisting illness (*2**,**6**,**31**,**36*).

Antimicrobial drug therapy for bacteremia and other extracolonic *C. difficile* infections varies greatly in the literature (*3**,**4*) because most infections have been polymicrobial in nature, and antimicrobial therapy has been directed at all organisms (*4**,**6*). Thus, optimal therapy for monomicrobial *C. difficile* bacteremia remains undefined. Intravenous vancomycin or metronidazole was frequently used in cases of bacteremia with well-described treatment regimens. Inappropriate antibacterial therapy for anaerobes, in general, appears to have serious consequences for patients (*32**,**35**,**37*). Based on in vitro antimicrobial drug susceptibility data, metronidazole may be the drug of choice for *C. difficile* bacteremia, although vancomycin was also effective in our patients. Clinical experiences in treating severe *C. difficile* infections were limited and mainly focused on *C. difficile*–associated diarrhea (*1**,**2**,**38*).

CDB, although uncommon, can be observed in persons with chronic underlying illness or coexisting gastrointestinal illnesses and is associated with high death rates. In the present study, a major finding was that the survival rate among patients who received appropriate antimicrobial therapy (either metronidazole or vancomycin) was higher than that among patients who received inappropriate antimicrobial drug therapy. Although multiple factorial conditions contributed to the high death rates among patients with CDB, insufficient therapeutic coverage for *C. difficile* may have contributed to the deaths of the patients. Early treatment of CDB with metronidazole or vancomycin may be helpful.

## References

[1] Aslam S, Hamill RJ, Musher DM. Treatment of *Clostridium difficile*–associated disease: old therapies and new strategies. Lancet Infect Dis. 2005;5:549–57. 10.1016/S1473-3099(05)70215-216122678

[2] Rupnik M, Wilcox MH, Gerding DN. *Clostridium difficile* infection: new developments in epidemiology and pathogenesis. Nat Rev Microbiol. 2009;7:526–36. 10.1038/nrmicro216419528959

[3] Garcia-Lechuz JM, Hernangomez S, Juan RS, Pelaez T, Alcala L, Bouza E. Extra-intestinal infections caused by *Clostridium difficile.* Clin Microbiol Infect. 2001;7:453–7. 10.1046/j.1469-0691.2001.00313.x11591212

[4] Jacobs A, Barnard K, Fishel R, Gradon JD. Extracolonic manifestations of *Clostridium difficile* infections. Presentation of 2 cases and review of the literature. Medicine (Baltimore). 2001;80:88–101. 10.1097/00005792-200103000-0000211307591

[5] Byl B, Jacobs F, Struelens MJ, Thys JP. Extraintestinal *Clostridium difficile* infections. Clin Infect Dis. 1996;22:712. 10.1093/clinids/22.4.7128729213

[6] Libby DB, Bearman G. Bacteremia due to *Clostridium difficile*–review of the literature. Int J Infect Dis. 2009;13:e305–9. 10.1016/j.ijid.2009.01.01419398213

[7] Saginur R, Fogel R, Begin L, Cohen B, Mendelson J. Splenic abscess due to *Clostridium difficile.* J Infect Dis. 1983;147:1105. 10.1093/infdis/147.6.11056854068

[8] Rampling A, Warren RE, Bevan PC, Hoggarth CE, Swirsky D, Hayhoe FG. *Clostridium difficile* in haematological malignancy. J Clin Pathol. 1985;38:445–51. 10.1136/jcp.38.4.4453857233PMC499176

[9] Feldman RJ, Kallich M, Weinstein MP. Bacteremia due to *Clostridium difficile*: case report and review of extraintestinal *C. difficile* infections. Clin Infect Dis. 1995;20:1560–2. 10.1093/clinids/20.6.15607548512

[10] Wolf LE, Gorbach SL, Granowitz EV. Extraintestinal *Clostridium difficile*: 10 years’ experience at a tertiary-care hospital. Mayo Clin Proc. 1998;73:943–7. 10.4065/73.10.9439787741

[11] McCabe WR, Jackson GG. Gram-negative bacteremia: I. Etiology and ecology. Arch Intern Med. 1962;110:847–55. 10.1001/archinte.1962.03620240029006

[12] Charlson ME, Pompei P, Ales KL, MacKenzie CR. A new method of classifying prognostic comorbidity in longitudinal studies: development and validation. J Chronic Dis. 1987;40:373–83. 10.1016/0021-9681(87)90171-83558716

[13] Knaus WA, Draper EA, Wagner DP, Zimmerman JE. APACHE II: a severity of disease classification system. Crit Care Med. 1985;13:818–29. 10.1097/00003246-198510000-000093928249

[14] Le Gall JR, Lemeshow S, Saulnier F. A new Simplified Acute Physiology Score (SAPS II) based on a European/North American multicenter study. JAMA. 1993;270:2957–63. 10.1001/jama.1993.035102400690358254858

[15] Yu VL, Chiou CC, Feldman C, Ortqvist A, Rello J, Morris AJ, An international prospective study of pneumococcal bacteremia: correlation with in vitro resistance, antibiotics administered, and clinical outcome. Clin Infect Dis. 2003;37:230–7. 10.1086/37753412856216

[16] Bone RC, Balk RA, Cerra FB, Dellinger RP, Fein AM, Knaus WA, Definitions for sepsis and organ failure and guidelines for the use of innovative therapies in sepsis. The ACCP/SCCM Consensus Conference Committee. American College of Chest Physicians/Society of Critical Care Medicine. Chest. 1992;101:1644–55. 10.1378/chest.101.6.16441303622

[17] Garner JS, Jarvis WR, Emori TG, Horan TC, Hughes JM. CDC definitions for nosocomial infections, 1988. Am J Infect Control. 1988;16:128–40. 10.1016/0196-6553(88)90053-32841893

[18] Baron EJ, Weissfeld AS, Fuselier PA, Brenner DJ. Classification and identification of bacteria. In: Murray PR, Baron EJ, Pfaller MA, Tenover FC, Yolken RH, editors. Manual of clinical microbiology, 6th ed. Washington: American Society for Microbiology Press; 1995. p. 249–64.

[19] Persson S, Torpdahl M, Olsen KE. New multiplex PCR method for the detection of *Clostridium difficile* toxin A (*tcdA*) and toxin B (*tcdB*) and the binary toxin (*cdtA/cdtB*) genes applied to a Danish strain collection. Clin Microbiol Infect. 2008;14:1057–64. 10.1111/j.1469-0691.2008.02092.x19040478

[20] Klaassen CH, van Haren HA, Horrevorts AM. Molecular fingerprinting of *Clostridium difficile* isolates: pulsed-field gel electrophoresis versus amplified fragment length polymorphism. J Clin Microbiol. 2002;40:101–4. 10.1128/JCM.40.1.101-104.200211773100PMC120100

[21] Spigaglia P, Mastrantonio P. Evaluation of repetitive element sequence-based PCR as a molecular typing method for *Clostridium difficile.* J Clin Microbiol. 2003;41:2454–7. 10.1128/JCM.41.6.2454-2457.200312791864PMC156551

[22] Clinical and Laboratory Standards Institute. Methods for antimicrobial susceptibility testing of anaerobic bacteria. Approved standard, 7th ed. M11–A7. Wayne (PA): The Institute; 2007.

[23] Bedimo R, Weinstein J. Recurrent extraintestinal *Clostridium difficile* infection. Am J Med. 2003;114:770–1. 10.1016/S0002-9343(03)00184-012829209

[24] Cid A, Juncal AR, Aguilera A, Regueiro BJ, Gonzalez V. *Clostridium difficile* bacteremia in an immunocompetent child. J Clin Microbiol. 1998;36:1167–8.954296510.1128/jcm.36.4.1167-1168.1998PMC104717

[25] Genta VM, Gilligan PH, McCarthy LR. *Clostridium difficile* peritonitis in a neonate. A case report. Arch Pathol Lab Med. 1984;108:82–3.6546347

[26] Studemeister AE, Beilke MA, Kirmani N. Splenic abscess due to *Clostridium difficile* and *Pseudomonas paucimobilis.* Am J Gastroenterol. 1987;82:389–90.3565349

[27] Spencer RC, Courtney SP, Nicol CD. Polymicrobial septicaemia due to *Clostridium difficile* and *Bacteroides fragilis.* Br Med J (Clin Res Ed). 1984;289:531–2. 10.1136/bmj.289.6444.5316432176PMC1442715

[28] Smith LD, King EO. Occurrence of *Clostridium difficile* in infections of man. J Bacteriol. 1962;84:65–7.1391432710.1128/jb.84.1.65-67.1962PMC277766

[29] Gerard M, Defresne N, Van der Auwera P, Meunier F. Polymicrobial septicemia with *Clostridium difficile* in acute diverticulitis. Eur J Clin Microbiol Infect Dis. 1989;8:300–2. 10.1007/BF019634552497002

[30] Elliott B, Reed R, Chang BJ, Riley TV. Bacteremia with a large clostridial toxin-negative, binary toxin-positive strain of *Clostridium difficile.* Anaerobe. 2009;15:249–51. 10.1016/j.anaerobe.2009.08.00619723585

[31] Benjamin B, Kan M, Schwartz D, Siegman-Igra Y. The possible significance of *Clostridium* spp. in blood cultures. Clin Microbiol Infect. 2006;12:1006–12. 10.1111/j.1469-0691.2006.01464.x16961638

[32] Rechner PM, Agger WA, Mruz K, Cogbill TH. Clinical features of clostridial bacteremia: a review from a rural area. Clin Infect Dis. 2001;33:349–53. 10.1086/32188311438901

[33] Pietrafitta JJ, Deckers PJ. Significance of clostridial bacteremia. Am J Surg. 1982;143:519–22. 10.1016/0002-9610(82)90206-96280507

[34] Balzan S, de Almeida Quadros C, de Cleva R, Zilberstein B, Cecconello I. Bacterial translocation: overview of mechanisms and clinical impact. J Gastroenterol Hepatol. 2007;22:464–71. 10.1111/j.1440-1746.2007.04933.x17376034

[35] Chen YM, Lee HC, Chang CM, Chuang YC, Ko WC. *Clostridium* bacteremia: emphasis on the poor prognosis in cirrhotic patients. J Microbiol Immunol Infect. 2001;34:113–8.11456356

[36] Kelly CP, Pothoulakis C, LaMont JT. *Clostridium difficile* colitis. N Engl J Med. 1994;330:257–62. 10.1056/NEJM1994012733004068043060

[37] Salonen JH, Eerola E, Meurman O. Clinical significance and outcome of anaerobic bacteremia. Clin Infect Dis. 1998;26:1413–7. 10.1086/5163559636872

[38] Bartlett JG. Narrative review: the new epidemic of *Clostridium difficile*–associated enteric disease. Ann Intern Med. 2006;145:758–64.1711692010.7326/0003-4819-145-10-200611210-00008

# Review article: *Clostridium difficile* Bacteremia, Taiwan1

**DOI:** 10.3201/eid1608.100064

**Authors:** Nan-Yao Lee, Yu-Tsung Huang, Po-Ren Hsueh, Wen-Chien Ko

**Affiliations:** Author affiliations: National Cheng Kung University Hospital and Medical College, Tainan, Taiwan (N.-Y. Lee, W.-C. Ko);; National Taiwan University Hospital and College of Medicine, Taipei, Taiwan (Y.-T. Huang, P.-R. Hsueh)

## Earning CME Credit

To obtain credit, you should first read the journal article. After reading the article, you should be able to answer the following, related, multiple-choice questions. To complete the questions and earn continuing medical education (CME) credit, please go to **http://cme.medscape.com/viewpublication/30063**. Credit cannot be obtained for tests completed on paper, although you may use the worksheet below to keep a record of your answers. You must be a registered user on Medscape.com. If you are not registered on Medscape.com, please click on the New Users: Free Registration link on the left hand side of the website to register. Only one answer is correct for each question. Once you successfully answer all post-test questions you will be able to view and/or print your certificate. For questions regarding the content of this activity, contact the accredited provider, CME@medscape.net. For technical assistance, contact CME@webmd.net. American Medical Association’s Physician’s Recognition Award (AMA PRA) credits are accepted in the US as evidence of participation in CME activities. For further information on this award, please refer to http://www.ama-assn.org/ama/pub/category/2922.html. The AMA has determined that physicians not licensed in the US who participate in this CME activity are eligible for *AMA PRA Category 1 Credits*™. Through agreements that the AMA has made with agencies in some countries, AMA PRA credit is acceptable as evidence of participation in CME activities. If you are not licensed in the US and want to obtain an AMA PRA CME credit, please complete the questions online, print the certificate and present it to your national medical association.

## Article Title: *Clostridium difficile* Bacteremia, Taiwan

### CME Questions

**1. You are seeing a 60-year-old woman who was admitted to the hospital for an infected foot ulcer. She has a history of poorly controlled diabetes, congestive heart failure, and stage III chronic kidney disease. Over the last 24 hours, she has developed fever and leukocytosis. Infection is suspected, but the source is not clear. The patient complains of fatigue and chills, but a review of systems is otherwise negative.**

**Based on data from the current study, which of the following statements regarding the possibility of *Clostridium difficile* bacteremia (CDB) in this patient is most accurate?**

A. Most CDB is found in young, healthy people

B. CDB usually develops in association with hospitalizations or stays in chronic care facilities

C. Abdominal pain is the most common presenting symptom of CDB

D. CDB does not occur without preceding diarrhea

**2. What was the most common source of bacteremia among patients in the current study?**

A. Primary bacteremia

B. Intra-abdominal infection

C. Bone and joint infection

D. Soft tissue infection

**3. The patient from question 1 is diagnosed with CDB. According to the current study, what is the best initial choice of antibiotic for her?**

A. Metronidazole

B. Imipenem

C. Ertapenem

D. Clindamycin

**4. The patient from question 1 is treated with an appropriate antibiotic. Which of the following statements regarding her prognosis is most accurate?**

A. Mortality is 75% regardless of antibiotic therapy

B. Use of broad-spectrum antibiotics reduces the risk for mortality to less than 10%

C. Survivors of CDB usually remain in the hospital for 3–5 days

D. Treatment with appropriate antibiotics can reduce the risk for mortality to 12.5%

## Activity Evaluation

**Table:** 

1. The activity supported the learning objectives.				
Strongly Disagree				Strongly Agree
1	2	3	4	5
2. The material was organized clearly for learning to occur.				
Strongly Disagree				Strongly Agree
1	2	3	4	5
3. The content learned from this activity will impact my practice.				
Strongly Disagree				Strongly Agree
1	2	3	4	5
4. The activity was presented objectively and free of commercial bias.				
Strongly Disagree				Strongly Agree
1	2	3	4	5
